# A study of diagnostic accuracy of the Florida Obsessive-Compulsive Inventory – Thai Version (FOCI-T)

**DOI:** 10.1186/s12888-015-0643-2

**Published:** 2015-10-14

**Authors:** Ratana Saipanish, Thanita Hiranyatheb, Sudawan Jullagate, Manote Lotrakul

**Affiliations:** Department of Psychiatry, Ramathibodi Hospital; Faculty of Medicine, Mahidol University, Mahidol, Thailand

**Keywords:** Florida Obsessive-Compulsive Inventory, Obsessive-compulsive disorder, Receiver operating characteristics (ROC), Sensitivity, Specificity, Thai

## Abstract

**Background:**

The Florida Obsessive-Compulsive Inventory (FOCI) is a self-reported measure to assess the symptoms and severity of obsessive-compulsive disorder (OCD), which can be completed in five minutes. Although preliminary studies have shown its good psychometric properties, the study of receiver operating characteristics (ROC) to use it as a screening tool has never been reported elsewhere. This study aimed to use the ROC analysis to determine the optimal cut-off score of the Thai version of the FOCI (FOCI-T).

**Methods:**

A total of 197 participants completed the FOCI-T, the Patient Health Questionnaire (PHQ-9), and the Pictorial Thai Quality of Life (PTQL), and they were also interviewed with the Mini International Neuropsychiatric Interview (MINI) for their diagnosis. The ROC analyses of the FOCI-T Severity Scores were computed to determine the best cut-off score.

**Results:**

When the Thai version of the MINI was used in the interview, it was found that 38 participants were diagnosed with OCD, 43 participants were non-OCD, and 116 participants were healthy adults. The ROC analyses indicated that the FOCI-T Severity Scale could significantly distinguish OCD patients from non-OCD patients and healthy adults. The area under curve was estimated to be 0.945 (95%CI = 0.903-0.972). A cut-off score of ≥5 provided the best sensitivity (0.92) and specificity (0.82).

**Conclusion:**

The Thai version of the Florida Obsessive-Compulsive Inventory has demonstrated its good predictive abilities, so it could be used as a brief screening tool to detect obsessive-compulsive disorder patients with high sensitivity and specificity.

## Background

Obsessive-compulsive disorder (OCD) is a debilitating disorder. It is characterized by the presence of recurrent, intrusive, and unwanted thoughts, impulses, or images which the patients attempt to eliminate, suppress, or neutralize with some other thoughts or actions which at last become repetitive ritualistic behaviors (e.g., hand or body washing, checking things, praying, counting, etc.). These symptoms often persist and increase over time, causing significant impairment in socialization, occupation, and other important areas of functioning including quality of life [1, 2].

There are a significant number (2-3 %) of individuals in the community who suffer from OCD [3, 4]. Some patients decide to seek professional assistance or treatment, but many patients do not, even though their symptoms are severe [3, 5]. Therefore, public health education campaigns and screening programs for OCD are needed to help the patients gain more timely access to treatment, with more ease and convenience. The screening tools for OCD would also be helpful in identifying OCD patients in the community, which, in turn, would benefit both patients and doctors.

The Florida Obsessive-Compulsive Inventory (FOCI) is a self-reported measure to assess the symptoms and severity of OCD. It can be easily completed in only five minutes which is very brief compared to other self-reported measures for OCD symptoms and severity [6, 7]. Although there are many well-developed self-reported measures of OCD, none of them is able to rapidly assess both symptom enumeration and severity in a simple format just like the FOCI does [6, 7]. The English version of the FOCI [6] was originally developed from the most acceptable measurement tool for symptom severity of OCD—the Yale-Brown Obsessive-Compulsive Scale (Y-BOCS)—and showed excellent psychometric properties in assessing the presence and severity of obsessive-compulsive symptoms. While very good psychometric properties of the FOCI have been shown in earlier studies [6–9], the data on a receiver operating characteristics (ROC) analysis to determine optimal diagnostic cut-off scores to use it as a screening tool for OCD have never been reported although they are needed [10]. Therefore, the present study aimed to assess the diagnostic accuracy of the Thai version of the FOCI by analyzing the ROC curve and cut-off scores, with the hope that the findings would yield support for subsequent uses of this instrument as a measure to identify the OCD patients in the Thai community.

## Methods

This study was approved by the Ethics Committee of the Faculty of Medicine, Ramathibodi Hospital, Bangkok. All participants provided their written informed consent before participating in the study.

### Participants

The study participants were recruited from psychiatric patients and healthy adults aged between 18 and 70 years old. Psychiatric patients were invited from the general psychiatric outpatient clinic and OCD clinic of the Department of Psychiatry, Ramathibodi Hospital, Bangkok. Healthy adults were recruited through community advertisement as well as solicitation of family members of hospital staffs and patients. The exclusion criteria were illiteracy, presence of intellectual disability, and psychosis.

### Measures

#### The Thai version of the Florida Obsessive-Compulsive Inventory (FOCI-T)

The FOCI consists of two scales: the Symptom Checklist and the Severity Scale [6]. On the Symptom Checklist, the patient would mark the presence (=1) or absence (=0) of common obsessions (10 items, e.g., Have you been bothered by unpleasant thoughts or images that repeatedly enter your mind such as: 1. Concerns with contamination (dirt, germs, chemicals, radiation) or acquiring a serious illness such as AIDS?, etc.) and compulsions (10 items, e.g., Have you ever felt driven to perform certain acts over and over again, such as: 1. Excessive or ritualized washing, cleaning or grooming?, etc. ). The total score of the Symptom Checklist is calculated by summing the scores of the presence of all items (range = 0–20), with higher scores indicating more symptoms. On the Severity Scale, the patient would rate the severity level (from 0 to 4) of endorsed symptoms on five items: time occupied, distress, degree of control, avoidance, and life influence. The total severity score is calculated by summing the scores of the five severity items (range = 0–20), with higher scores corresponding to greater symptom severity. In the English version, the FOCI demonstrates strong internal consistency for the Symptom Checklist (Kuder-Richardson-20 (KR-20) = 0.83) and the Severity Scale (Cronbach’s alpha (α) = 0.89). Concurrent validity has been reported with very strong correlations between the FOCI Severity Scale and the clinician-rated measures of OCD symptom severity (rs >0.8). When comparing the Thai version of the FOCI to the English version, it could be seen that the Thai version of the Florida Obsessive-Compulsive Inventory (FOCI-T) has also shown good internal consistency reliability (KR-20 = 0.86 for the Symptom Checklist and α = 0.92 for the Severity Scale) and satisfactory concurrent validity associated with the Thai version of the Yale-Brown Obsessive-Compulsive Scale-Second Edition (rs > 0.9) [7].

### The Pictorial Thai Quality of Life (PTQL)

The PTQL is a self-reported tool to measure the six domains of quality of life of the Thai people: Physical, Cognitive, Affective, Social Function, Economic, and Self-Esteem. It consists of 25 items, all of which have sufficient discriminant power. The possible total scores range from 0 to 75, with higher scores reflecting better quality of life of the person. The PTQL has demonstrated a high level of concurrent validity (Pearson’s correlation coefficient = 0.92) and excellent internal consistency (α = 0.88) [11]**.**

### The Patient Health Questionnaire (PHQ-9)

The PHQ-9 [12] is a self-reported measure, consisting of nine questions based on the DSM-IV criteria for major depressive episode. It has the potential to grade depressive severity, which refers to symptoms experienced by the patients during the period of two weeks prior to answering the questionnaires. Each item has a 4-point scale: 0 = ‘not at all’, 1 = ‘several days’, 2 = ‘more than half of the days’, and 3 = ‘nearly every day’. The total score is calculated by summing the scores of all nine items (range = 0–27), with higher scores indicating greater depressive severity. The Thai version of the PHQ-9 has satisfactory internal consistency (Cronbach’s alpha = 0.79) and moderate convergent validity (r = 0.56) [13].

### Mini International Neuropsychiatric Interview (MINI)

The MINI [14] is a structured clinical diagnostic interview for DSM-IV Axis-I psychiatric disorders. It consists of various modules which cover a wide range of psychiatric disorders. It has high reliability and validity and can be reliably administered by interviewers who have appropriate training. This study used the Thai version of MINI [15], which was translated from the MINI, version 5. It showed high specificity, negative predictive value, and efficiency for every diagnosis (>0.81), with a specificity of 0.95, negative predictive value of 0.97, and efficiency of 0.92 for the diagnosis of current obsessive-compulsive disorder. The third author (SJ), who was a clinical psychologist, underwent training to use this instrument.

### Study design

**Fig. 1:**
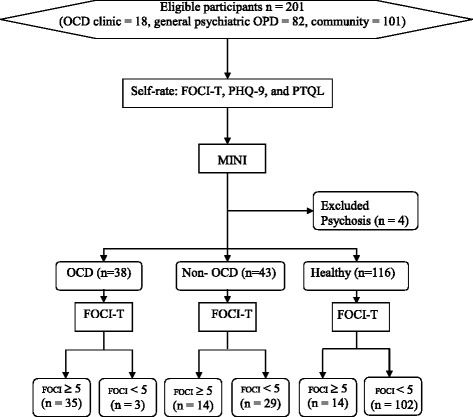
Flowchart of this diagnostic accuracy study of the FOCI-T

After receiving an explanation on the objectives and the process of the study, all participants who were interested in participating in the study on a voluntary basis provided their informed consent and then completed the FOCI-T, PHQ-9, and PTQL. After that, the third author (SJ), a clinical psychologist who was blinded to all aforementioned measurement scores and the sources of participants, interviewed them for their diagnosis by using the Thai version of the MINI. The participants were divided into three study groups, which were OCD, non-OCD, and healthy as in the flowchart (Fig. 1). With regard to the OCD group, the patients had to be diagnosed with a positive result on the obsessive-compulsive module in the Mini International Neuropsychiatric Interview (MINI), while those in the non-OCD group had to have at least one other psychiatric diagnosis and have a negative result on the obsessive-compulsive module in the MINI. Finally, to be qualified as healthy, the participants were required to have no current disorders as confirmed with the use of all modules in the MINI.

### Statistical analyses

The collected data were analyzed using the Statistical Package for the Social Science 18.0 (SPSS 18.0). Independent sample t-tests were used with continuous variables to compare the mean scores between groups. Chi-square tests were also employed with categorical variables, and analyses of variances were applied with continuous variables to examine the differences among the groups.

The diagnostic accuracy of the FOCI-T Severity Scale was analyzed with the MedCalc version 8.0. To determine the best cut-off score, the indices of sensitivity and specificity were used along with the receiver operating characteristic (ROC) curve. Positive-likelihood ratios (sensitivity/(1–specificity)), negative-likelihood ratios ((1–sensitivity)/specificity), positive predictive value (PPV), and negative predictive value (NPV) of the test were analyzed concurrently.

## Results

Two hundred and one eligible participants from three sources (OCD clinic = 18, general psychiatric outpatient clinic = 82, and community = 101) participated in the study on voluntary basis. Since four participants were excluded after being diagnosed with psychosis by using the MINI interview, a total of 197 participants completed the entire process. When being interviewed with the use of the Thai version of the MINI, most of the patients from the OCD clinic were found to have OCD (OCD = 83.3 %), while most of the healthy participants did not (OCD = 1 %). Meanwhile, some of the patients from the general psychiatric outpatient clinic (28 %) had OCD. In total, 38 participants were diagnosed with OCD, 43 participants were non-OCD, and 116 participants were qualified as healthy adults, as shown in Table 1. The MINI diagnoses also revealed comorbidities of the participants in the OCD group, which were mood disorders (16/38), anxiety disorders (21/38), eating disorders (1/38), substance use disorders (5/38), and suicidality (9/38). On the other hand, the comorbidities of those in the non-OCD group were mood disorders (17/43), anxiety disorders (9/43), eating disorders (4/43), substance use disorders (6/43), and suicidality (23/43). The characteristics of the participants in the OCD group, non-OCD group, and healthy adults are presented in Table 2. There were no differences among the three groups of participants in terms of age, gender ratio, marital status, and educational background.

**Table 1:** MINI diagnosis of 197 participants from OCD clinic, general psychiatric outpatient clinic, and community

MINI diagnosis	Sources of participants			Total
	OCD clinic (*n* = 18)	General psychiatric outpatient clinic (*n* = 78)	Community (*n* = 101)	
OCD (%)	15 (39.5)	22 (57.9)	1 (2.6)	38 (100)
Comorbid				
–Mood disorders (%)	9 (56.2)	7 (43.8)	-	16 (100)
–Anxiety disorders (%)	10 (47.6)	11 (52.4)	-	21 (100)
–Eating disorders (%)	1 (100)	-	-	1 (100)
–Substance disorders (%)	2 (40)	3 (60)	-	5 (100)
–Sucidality (%)	4 (44.4)	5 (55.6)	-	9 (100)
Non-OCD (%)	1 (2.3)	34 (79.4)	8 (18.6)	43 (100)
Mood disorders (%)	-	17 (100)	-	17 (100)
Anxiety disorders (%)	-	7 (77.8)	2 (22.2)	9 (100)
Eating disorders (%)	-	1 (25.0)	3 (75.0)	4 (100)
Substance disorders (%)	-	6 (100)	-	6 (100)
Sucidality (%)	1 (4.3)	19 (82.6)	3 (13.0)	23 (100)
Healthy (%)	2 (1.7)	22 (19.0)	92 (79.3)	116 (100)

Note: *MINI* the Mini International Neuropsychiatric Interview, *OCD* Obsessive-compulsive disorder

**Table 2:** Characteristics of participant groups

	OCD (*n* = 38)	Non-OCD (*n* = 43)	Healthy (*n* = 116)	F / ϰ^2^	p
Female (%)	15 (39.5)	21 (48.8)	65 (56.0)	3.272	0.19
Age (SD)	33.74 (14.37)	40.37 (14.06)	40.13 (17.06)	2.515	0.08
Marital Status				4.234	0.83
Single (%)	23 (60.5)	28 (65.1)	67 (57.8)		
Married (%)	14 (36.8)	13 (27.7)	42 (36.2)		
Level of education				10.752	0.71
≤ Secondary school (%)	10 (26.3)	9 (21.0)	20 (17.2)		
≥ Graduated (%)	27 (71.1)	34 (79.0)	94 (81.0)		
FOCI-T					
Number of symptoms (SD)	8.57 (4.38)^a, c^	3.60 (3.59)^d^	2.09 (2.55)	58.455	<0.0001
Severity Score (SD)	10.50 (4.17)^a, c^	3.35 (3.43)^e^	1.47 (2.58)	118.748	<0.0001
PHQ-9 (SD)	9.31 (6.55)^b, c^	6.44 (4.91)^e^	2.92 (2.93)	35.105	<0.0001
PTQL (SD)	34.23 (11.90)^b, c^	40.69 (13.21)^e^	51.91 (12.33)	33.974	<0.0001

*OCD* Obsessive-compulsive disorder, *FOCI-T* the Florida Obsessive-Compulsive Inventory, *PHQ-9* the Patient Health Questionnaire, *PTQL* the Pictorial Thai Quality of Life

^a^ *p* <0.0001 OCD vs non-OCD

^b^ *p* <0.05 OCD vs non-OCD

^C^ *p* < 0.0001 OCD vs healthy

^d^ *p* < 0.005 non-OCD vs healthy

^e^ *p* < 0.0001 non-OCD vs healthy

As shown in Table 2, there were significant differences in numbers of symptoms and severity scores of the FOCI-T among the three groups of participants. The OCD group had a significantly higher number of symptoms and higher severity scores than the non-OCD and healthy groups. Although the severity score of the FOCI-T of the non-OCD group was not much higher than that of the healthy group, it showed a significant difference. This pattern was also found in the PHQ-9, which demonstrated depression severity. The group which had the highest PHQ-9 score was the OCD group, followed by the non-OCD and healthy groups, respectively. As regards quality of life assessed with the PTQL, the OCD group had the lowest score with significant differences from those of the non-OCD and healthy adult groups.

### FOCI-T discrimination of OCD

**Fig. 2:**
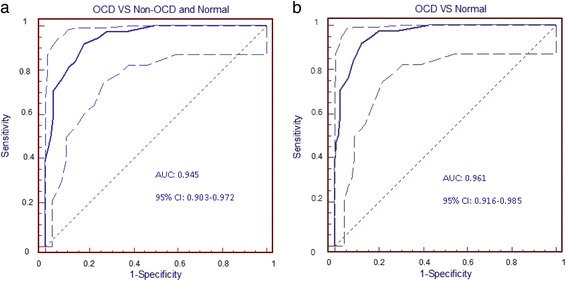
The ROC curve (with 95 % confidence interval) of the FOCI-T Severity Scale discriminating (**a**) OCD versus non-OCD and Helathy and (**b**) OCD versus Healthy

As for ROC analyses, the scores from the FOCI-T Severity Scale were calculated. It was illustrated by the ROC curve that the FOCI-T Severity Scale performed well in distinguishing participants with OCD from a mixed group of non-OCD patients and healthy adults (Fig. 2). The area under the curve (AUC) was 0.945 (SE = 0.026, 95 % CI: 0.903 0.972,*p* = 0.0001). Table 3 demonstrates the sensitivity, specificity, PPV, NPV, and positive and negative likelihood ratios for different thresholds in diagnosing OCD. At a cut-off score of ≥5, the FOCI-T Severity Scale yielded the best sensitivity (0.92) and specificity (0.82) to discriminate OCD from non-OCD and healthy adults. Based on our sample, the percentage of OCD was 19.3 %. The PPV and NPV were 0.56 and 0.98, respectively, for detection of OCD.

**Table 3:** The performance of FOCI-T Severity cut-off scores in detecting OCD

Cut-off score	Sensitivity	95 % CI	Specificity	95 % CI	+LR	-LR	PPV	NPV
Discrimination of OCD from non-OCD and healthy adults								
≥3	0.97	0.86 – 0.99	0.72	0.65 – 0.79	3.52	0.04	0.46	0.99
≥4	0.95	0.82 – 0.99	0.76	0.69 – 0.83	3.96	0.07	0.48	0.98
≥5 *	**0.92**	**0.79 – 0.98**	**0.82**	**0.76 – 0.88**	**5.23**	**0.10**	**0.56**	**0.98**
≥6	0.84	0.69 – 0.94	0.86	0.80 – 0. 91	6.09	0.18	0.59	0.96
≥7	0.82	0.66 – 0.92	0.89	0.83 – 0.94	7.63	0.21	0.64	0.95
≥8	0.76	0.60 – 0.89	0.92	0.87 – 0.96	10.11	0.26	0.71	0.94
≥9	0.71	0.54 – 0.85	0.96	0.92 – 0.99	18.83	0.30	0.82	0.93
≥10	0.61	0.43 – 0.76	0.96	0.92 – 0.99	16.04	0.41	0.79	0.91
Discrimination of OCD from healthy adults								
≥3	0.97	0.86 – 0.99	0.80	0.72 – 0.87	4.91	0.03	0.62	0.99
≥4	0.95	0.82 – 0.99	0.83	0.76 – 0.89	5.78	0.06	0.65	0.98
≥5 *	**0.92**	**0.79 – 0.98**	**0.88**	**0.81 – 0.93**	**7.63**	**0.09**	**0.71**	**0.97**
≥6	0.84	0.69 – 0.94	0.91	0.85 – 0.96	9.77	0.17	0.76	0.95
≥7	0.82	0.66 – 0.92	0.92	0.86 – 0.96	10.51	0.20	0.77	0.94

*+LR* Positive likelihood ratio, *−LR* Negative likelihood ratio, *PPV* Positive predictive value, *NPV* Negative predictive value. * *and bold data *Highest area under the ROC curve with a significance level (*p*=0.0001)

To distinguish OCD patients from a group of healthy adults, the ROC curve analysis was also performed. The AUC was 0.961 (SE = 0.022, 95 % CI: 0.916 0.985,*p* = 0.0001). A FOCI-T Severity Scale cut-off score of ≥5, the same point as the aforementioned result, also yielded the best sensitivity (0.92), specificity (0.88), PPV (0.71), and NPV (0.97), as shown in the latter part of Table 3.

## Discussion

This study aimed to use the ROC analysis to determine the best cut-off score of the Thai version of the FOCI (FOCI-T). Our results indicated that the cut-off score of ≥5 of the FOCI-T Severity Scale could distinguish OCD patients from both a mixed group of non-OCD patients and healthy adults and a group of healthy adults with the optimal sensitivity and specificity. The ROC analyses were performed to distinguish the OCD patients from both groups (the mixed group and healthy group) so that the FOCI-T can be subsequently used in various settings of the Thai community, which may comprise only healthy people or a mixture between healthy people and people with other psychiatric disorders. It is worth noting that only the FOCI-T Severity Scale, not the Symptom Checklist, was used to analyze the ROC curve and cut-off scores because a large number of people have some symptoms similar to OCD, such as doubting and checking if one had turned off the switch or locked the car, but their symptoms were not severe enough to be OCD [3, 16]. Therefore, the FOCI-T Severity Scale, which assesses distress, disturbance, and life influence of the OCD symptoms, was deemed appropriate to be analyzed and used with the specified cut-off score.

On the website of the FOCI, http://www.ocdscales.org/index.php?page=scales [17], it is suggested that individuals should consider seeking professional consultation from specialists for treatment of OCD if their resulting score of the FOCI Severity Scale is ≥8, which is higher than the finding reported in the present study. However, the FOCI website does not specify why the cut-off score of ≥8 was chosen, nor does it supply information on sensitivity and specificity. According to a review conducted by Overduin and Furnham [10], there has been no previous study on the ROC analysis for the FOCI. To our knowledge, this study is the first ROC analyses of the FOCI. At the cut-off score of ≥8 in our result shown in Table 3, the specificity was very high (0.92) but the sensitivity was 0.76, which indicated that this cut-off score might reflect a more definite OCD case that truly required professional help. However, our purpose was to develop the FOCI-T to be a screening tool, so a measure with a higher sensitivity is needed [18]. Therefore, a cut-off score of the FOCI-T severity Scale of 5 or greater is considered sufficiently appropriate.

This study aimed to examine the sensitivity and specificity of the FOCI-T so that it could be used as a screening tool for OCD patients with impaired functioning. To assure that the OCD patients in this study were active cases with impairment of functions, we used the Mini International Neuropsychiatric Interview (MINI) which was considered a gold standard for a diagnosis of OCD with impaired functions within the past month. The obsessive-compulsive module in the Thai version of the MINI used in our study has a very high specificity (0.95) which is useful for ‘ruling in’ the disorder if a person has a positive result, and a high negative predictive value (0.97) which means a 97 % chance of not having OCD if a person has a negative result [19].

Our results have also demonstrated that the non-OCD patients and healthy adults can sometimes have symptoms of depression and obsession-compulsion as mentioned by Salkovskis [16], but their symptoms are not severe enough to be diagnosed as depressive disorders or OCD. This may explain why some participants in the non-OCD and the healthy adult groups have scored on the PHQ-9 and FOCI-T, though significantly lower than the OCD group. In addition, the OCD group had highest score on the PHQ-9 and lowest score on the PTQL which demonstrated that the OCD group had significantly more severe depression and lower quality of life, which could mean more severe psychopathology, compared to the non-OCD and healthy adult groups. Such findings are consistent with the findings of a previous study carried out by Subramamian et al. [20] which has reported poorer quality of life in the patients with OCD compared to patients with other mental illnesses or physical illnesses.

Although our study was undertaken during the transition from DSM-IV-TR to DSM-5, there was no significant change in the DSM-5 diagnostic criteria for OCD, other than the addition of the “insight” specifier to distinguish between individuals with good or poor insight of the disorder [21, 22]. Therefore, the diagnosis of OCD done with the use of the MINI in this study was appropriate and the FOCI-T will continue to be a useful tool in the time of the DSM-5 era. The main limitation was that the study was conducted in a hospital, so the OCD patients recruited may not represented the characteristics of the OCD patients in the community. However, we consider this study to be an important first step to pave the way for future studies on OCD in communities and to subsequently improve the FOCI-T to better suit the community population. Another limitation was that all MINI interviews were conducted by a single rater, and this could result in some errors. However, this interviewer has been well-trained to use the Thai version of the MINI and her interrater reliability (0.91) has been reported elsewhere [23]. Lastly, the FOCI used in this study was in the Thai language, which might be used only for Thai people. Therefore, studies carried out with the FOCI in other languages are needed.

## Conclusion

The Florida Obsessive-Compulsive Inventory (FOCI) is a useful measure to assess the symptoms and severity of obsessive-compulsive disorder. As evident by the results of the present study, the Thai version of the FOCI is also found to be a tool to identify obsessive-compulsive disorder patients from non-OCD patients and healthy adults that would be useful as a screening tool for OCD in the Thai community. A cut-off score of the FOCI Severity Scale of ≥5, which provided the best sensitivity and specificity, is recommended to be used as a screening tool for OCD.

## Abbreviations

AUC: Area under the (receiver operating characteristics) curve
CI: Confidence interval
FOCI: The Florida Obsessive-Compulsive Inventory
FOCI-T: The Thai version of the FOCI
KR-20: Kuder-Richardson-20
+LR: Positive likelihood ratio
−LR: Negative likelihood ratio
MINI: The Mini International Neuropsychiatric Interview
NPV: Negative predictive value
OCD: Obsessive-compulsive disorder
PHQ-9: The Patient Health Questionnaire
PPV: Positive predictive value
PTQL: The Pictorial Thai Quality of Life
ROC: Receiver operating characteristics
SE: Standard error
SPSS 18.0: The Statistical Package for the Social Science 18.0
YBOCS: The yale-brown obsessive-compulsive scales
